# Aging out of Place: Factors Related to Quality of Life Among Older Refugees in the United States

**DOI:** 10.1093/geroni/igab046.912

**Published:** 2021-12-17

**Authors:** Jonix Owino, Heather Fuller

**Affiliations:** 1 North Dakota State University, Horace, North Dakota, United States; 2 North Dakota State university, Fargo, North Dakota, United States

## Abstract

Refugees flee their home countries, migrating to countries such as the US for safety. The psychological distress they experience may compromise their adaptation and well-being. However, little is known about quality of life among aging refugees who migrate to the US as adults, and in particular whether quality of life varies among refugees by sociodemographic factors such as age, sex, country of origin, and length of residence. Moreover, limited research exists examining the role of social connectedness for aging refugees’s quality of life. The current study explores sociodemographic and social connection factors associated with quality of life among aging refugees (N = 108; aged 50+). Refugees from Bhutan, Burundi, and Somalia were recruited from a Midwestern small city to complete an in-depth survey assessing social factors and well-being. Hierarchical regression analyses showed that females, older individuals, and African refugees reported lower quality of life, while length of residence was not associated with quality of life. When controlling for sociodemographic factors, greater social integration and lower loneliness were significantly associated with higher quality of life. There was also a significant interaction between loneliness and sex in predicting quality of life, indicating that greater loneliness was associated with reduced quality of life for women but not men. Study findings will be discussed in light of cultural variations within refugee groups and with the goal of highlighting ways to best support aging refugees’ well-being and develop social programs that can effectively cater to issues of aging among refugees.

